# Taxonomic re-evaluation of African anuran trypanosomes with the redescription and molecular diagnosis of *Trypanosoma* (*Trypanosoma*) *nelspruitense* Laveran, 1904 and *Trypanosoma* (*Haematomonas*) *grandicolor* Pienaar, 1962

**DOI:** 10.1017/S0031182023000203

**Published:** 2023-05

**Authors:** Bernard J. Jordaan, Louis H. du Preez, Edward C. Netherlands

**Affiliations:** 1 African Amphibian Conservation Research Group, Unit for Environmental Sciences and Management, North-West University, Potchefstroom, South Africa; 2 South African Institute for Aquatic Biodiversity, Somerset Street, Makhanda 6140, South Africa; 3 Department of Zoology and Entomology, University of the Free State, PO Box 339, Bloemfontein 9300, South Africa

**Keywords:** *Amietia delalandii*, blood parasite, frog, molecular characterization, morphometrics, phylogeny, *Xenopus laevis*

## Abstract

The aquatic and terrestrial clades of species of *Trypanosoma* could provide insight into the evolutionary history of the genus, as well as complementary information for biomedical studies of medically and economically important species of *Trypanosoma*. The ecological interactions and phylogeny of aquatic trypanosomes are currently not well-understood, mostly due to their complex life cycles and a deficiency of data. The species of *Trypanosoma* from African anuran hosts are of the least understood taxa in the genus. Trypanosomes were collected from South African frogs and subjected to morphological and phylogenetic analyses. This study redescribes *Trypanosoma* (*Trypanosoma*) *nelspruitense* Laveran, 1904 and *Trypanosoma* (*Haematomonas*) *grandicolor* Pienaar, 1962, with morphological and molecular data. The present study aims to create a platform for further future research on African anuran trypanosomes.

## Graphical abstract

**Figure:**
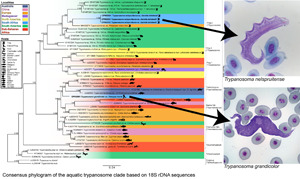

## Introduction

The genus *Trypanosoma* Gruby, 1843 (Euglenozoa: Kinetoplastea: Trypanosomatidae) is a globally occurring group of unicellular obligate haemoparasites (Netherlands *et al*., 2014). Trypanosomes are typically recognized by the presence of an undulating membrane and kinetoplast; however, they vary morphologically from elongated to round body shapes, even within a single species (d'Avila-Levy *et al*., 2015; Attias *et al*., 2016). Trypanosomes are known to parasitize all the vertebrate classes (i.e. fishes, amphibians, reptiles, birds and mammals) and are an ancient group of parasites. Fossil records of trypanosomes have been found trapped within amber in triatomine feces from approximately 15–45 mya (Poinar, 2005), as well as a sister-genus, *Paleoleishmania* (Poinar and Poinar, 2004), being reported from the abdominal midgut of a fossilized sand fly dating at least 100 mya (Poinar and Poinar, 2004). The invertebrate vectors of trypanosomes include dipterans (e.g. mosquitoes, biting midges and hippoboscid louse flies), triatomine bugs and leeches (Ramos and Urdaneta-Morales, 1977), in which the trypomastigotes typically undergo complex development, transforming into several different stages before infecting a new host (Magez *et al*., 2021; Vanhove *et al*., 2022). Generally, trypanosomes alternate between infecting a vertebrate and invertebrate host during their lifecycles (Hamilton *et al*., 2007). Trypanosomes are of considerable scientific importance, as this group is known to cause several diseases, such as African trypanosomiasis and Chagas disease, in humans and livestock. The majority of scientific research has concentrated on trypanosomes that are pathogenic to humans and livestock; however, relatively little is known about the species of *Trypanosoma* parasitizing other host animals (Hughes and Piontkivska, 2003; Dvořáková *et al*., 2015; Attias *et al*., 2016; Bernal and Pinto, 2016). The availability of data specifically for trypanosomes infecting anurans is lacking (Leal *et al*., 2009; Netherlands *et al*., 2014; Dvořáková *et al*., 2015; Attias *et al*., 2016; Bernal and Pinto, 2016).

The study of anuran trypanosomes played an important historical role in developing an understanding of the genus of *Trypanosoma*. Trypanosomes were first discovered by Gluge (1842) in a frog host species but were not named. The first description of trypanosomes was provided by Mayer (1843) from the blood of the European frog, *Pelophylax esculentus* Linnaeus, 1758 (syn. *Rana esculenta*), although he initially incorrectly identified the parasites, describing the organisms as *Trypanosoma loricatum* (Mayer, 1843) Dutton, Todd and Tobey, 1907 (syn. *Paramaecium loricatum*) and *Trypanosoma rotatorium* (Mayer, 1843) Laveran and Mesnil, 1901 (syn. *Amoeba rotatoria*). From an infection in the blood of an unidentified European frog host species, the genus of *Trypanosoma* was created by Gruby (1843) with the description of the type species, *Trypanosoma sanguinis* Gruby, 1843. However, it has since been proposed by others that Gruby (1843) described a mixed infection of multiple trypanosome species, and that *T. sanguinis* is a junior synonym of *T. rotatorium* (Laveran and Mesnil, 1901, 1907; Diamond, 1965; Spodareva *et al*., 2018).

In Africa, most of the species of *Trypanosoma* from anurans were recorded early in the 20th century. Only 14 recognized species of *Trypanosoma* have been reported from anurans in Africa (Table 1; Laveran, 1904; Bardsley and Harmsen, 1973; Dvořáková *et al*., 2015; Fermino *et al*., 2019). This is surprising considering the geographical scale and rich biodiversity of the African continent. Furthermore, only 2 species, *Trypanosoma nelspruitense* Laveran, 1904 and *Trypanosoma grandicolor* Pienaar, 1962, have been described from South African anurans, infecting the common river frog (*Amietia delalandii* Duméril and Bibron, 1841) and the African clawed frog (*Xenopus laevis* Daudin, 1802), respectively. These species descriptions severely lack morphological metrics, with no molecular data available. No records of *T. nelspruitense* or *T. grandicolor* have been reported since their original descriptions; therefore, this study will be the only report of these 2 species other than their original records. Fantham *et al*. (1942) briefly mention having observed rounded *T. rotatorium* infections in *X. laevis* and *Amietia fuscigula* (syn. *Rana fuscigula*) from South Africa. Several unnamed trypanosomes were also reported from South African anurans in the study of Netherlands (2019), from grass frogs (*Ptychadena anchietae*), leaf-folding frogs (*Afrixalus delicatus* and *A. fornasini*), reed frogs (*Hyperolius argus*, *H. marmoratus*, *H. tuberilinguis*), shovel-nosed frogs (*Hemisus marmoratus*), tree frogs (*Leptopelis natalensis* and *L. mossambicus*), puddle frogs (*Phrynobatrachus mababiensis*) and toads (*Sclerophrys garmani* and *S. gutturalis*).

**Table 1.** African anuran *Trypanosoma* index. Type hosts, type localities, and original description authorities in bold.

Species of *Trypanosoma*	African record	Author	Known host species	Known vector species
*T. bocagei*	**Guinea-Bissau**	**França (1911)**	***Sclerophrys regularis* (syn. *Bufo regularis*)**	
	Tunisia	Lebailly and Caillon (1919)	*Sclerophrys mauritanica* (syn. *Bufo mauritanicus*)	
*T. elegans*	Congo	Martin *et al*. (1909)	*Sclerophrys regularis* (syn. *Bufo regularis*)	
*T. grandicolor*	**South Africa: Potchefstroom**	**Pienaar (1962)**	***Xenopus laevis***	
*T. inopinatum*	**Algeria: Dra-el-Mizan (Kabylie)**	**Sergent and Sergent (1904,** 1905**)**	***Pelophylax esculentus* (syn. *Rana esculenta*)**	*Helobdella algira* (Desser *et al*., 1973)
	Algeria	Billet (1904)		
*T. karyozeukton*	**Gambia: Cape St. Mary**	**Dutton and Todd (1903)**	**Frog (unknown species)**	
*T. loricatum*	Congo	Dutton *et al*. (1907)	*Amnirana galamensis* (syn. *Rana galamensis*) *Hyperolius marmoratus* (syn. *Rappia marmorata*) *Ptychadena mascareniensis* (syn. *Rana mascariensis*) *Ptychadena oxyrhynchus* (syn. *Rana oxyrhynchus*) *Sclerophrys regularis* (syn. *Bufo regularia*)	
*T. mega*	Angola	França (1925)	*Ptychadena oxyrhynchus* (syn. *Rana oxyrhynchus*) *Sclerophrys regularis* (syn. *Bufo regularis*) *Tomopterna tuberculosa* (syn. *Rana tuberculosa*) **Frog (unknown species)**	
	Congo	Broden (1905) Rodhain (1907) Kerandel (1909) Martin *et al*. (1909) Schwetz (1944)		
	**Gambia: MacCarthy Island**	**Dutton and Todd (1903)**		
	Nigeria	MacFie (1914)		
	Africa (unspecified)	Walton (1947)		
*T. nelspruitense*	**South Africa: Mbombela (formerly Nelspruit)**	**Laveran (1904)**	***Amietia delalandii* (syn. *Rana angolensis* and *Rana theileri*)**	
*T. parroti*	**Algeria**	**Brumpt (1923)**	***Discoglossus pictus***	
*T. rotatorium*	Congo	Rodhain (1907) Martin *et al*. (1909) Schwetz (1930, 1944) Pérez-Reyez (1967)	*Alytes obstetricans* (syn. *Bufo obstetricans*) *Amietia fuscigula* (syn. *Rana fuscigula*) *Hoplobatrachus occipitalis* (syn. *Rana occipitalis*) *Hyperolius sp.* *Leptodactylus albilabris* (syn. *Rana albilabris*) *Leptopelis* sp. *Ptychadena mascareniensis* (syn. *Rana mascariensis*) *Ptychadena oxyrhynchus* (syn. *Rana oxyrhynchus*) *Ptychadena perreti* *Sclerophrys regularis* (syn. *Bufo regularis*) *Xenopus laevis* Frog (unknown species) Toad (unknown species)	*Culex territans* (Desser *et al*., 1973) *Aedes aegypti* (Ramos & Urdaneta-Morales, 1978) *Helobdella algira* (Desser *et al*., 1973)
	Egypt	Mohammed and Mansour (1959, 1966)		
	Guinea-Bissau	Holberton (1966)		
	Nigeria	MacFie (1914) Lloyd *et al*. (1924)		
	South Africa	Fantham *et al*. (1942)		
	Sudan	Balfour (1908) Stevenson (1911)		
	Uganda: Bugala Island	Hoare (1932)		
	Africa (unspecified)	Rousselot (1953)		
*T. sanguinis*	Gambia	Dutton and Todd (1903)	*Ptychadena trinodis* (syn. *Rana trinodis*)	
*T. sergenti*	**Algeria**	**Brumpt (1923)**	***Discoglossus pictus***	
*T. somalense*	**Somalia**	**Brumpt (1906)**	***Sclerophrys regularis* (syn. *Bufo reticulatus*)**	
*T. tumida*	**Burundi: Bujumbura (formerly Usumbura), Tanganyika Territory**	**Avérinzev (1916)**	***Amietia nutti* (syn. *Rana nutti*)**	

Leal *et al*. (2009) proposed that the dissonance in the characterization of species of *Trypanosoma* is due to the extreme polymorphism that may be found in a single species, as well as individual species being able to infect numerous vertebrate and invertebrate host species. Furthermore, they believe that the absence of molecular and life cycle ecological data in species descriptions up until recent years has led to numerous taxonomic errors; thus, species described separately in different hosts or geographical areas could be synonymous (Leal *et al*., 2009; Hayes *et al*., 2014). Accordingly, the widespread inclusion of molecular data in non-human trypanosome research, as well as revisiting existing described species, would be of great scientific importance in order to achieve better taxonomic organization in the genus and a better ecological understanding of all the involved host species.

The most significant division in the phylogenetic relationships of non-human trypanosomes is between the species infecting terrestrial and aquatic vertebrates (Hamilton *et al*., 2007; Fermino *et al*., 2019). Based on phylogenetic analyses, amphibian and fish trypanosomes are the origin from which all other trypanosomes evolved, according to Hamilton *et al*. (2007). Because anurans are exposed to both aerial and aquatic trypanosome vectors, they could be a link between the 2 trypanosome clades (Spodareva *et al*., 2018), providing insight into other species of *Trypanosoma* (Bernal and Pinto, 2016). Thus, anuran trypanosomes are possibly the key to understanding the evolutionary history of the entire genus. Due to a lack in molecular data for this group in general and no molecular data for any species of anuran trypanosomes from South Africa, it is important to research the trypanosomes infecting frogs.

The aim of this study was to provide a basis for future taxonomic work on amphibian trypanosomes from Africa. Thus, the objectives of the present study were to (1) revise the original descriptions as well as provide redescriptions of the only 2 currently recognized species of anuran trypanosomes described from South Africa, *T. nelspruitense* and *T. grandicolor*; (2) provide the first molecular data for these species; (3) conduct a phylogenetic analysis on these 2 species of *Trypanosoma* from South Africa. This would provide a platform for future research on trypanosomes within South Africa and would contribute to a better understanding of the genus *Trypanosoma*, as this species is part of the aquatic trypanosome clade, from which all other trypanosome species potentially originated. Herein, we redescribe *T. nelspruitense* and *T. grandicolor*, with morphological and molecular data. These 2 species are placed within monophyletic subclades of the aquatic trypanosome clade. The present study follows the taxonomic classification system of *Trypanosoma* subgenera proposed by Kostygov *et al*. (2021), placing the species *T. nelspruitense* and *T. grandicolor* in the subgenera *Trypanosoma* Gruby, 1843 and *Haematomonas* Mitrophanow, 1883, respectively.

## Materials and methods

### Collection of research specimens

*Amietia delalandii*, the type host of *T. nelspruitense*, was 1 of 2 host species targeted for the present study. The second host targeted was *X. laevis*, the type host of *T. grandicolor*. In June 2020, *A. delalandii* were sampled by hand from 2 sites in Mbombela (formerly Nelspruit), Mpumalanga, South Africa, the type locality of *T. nelspruitense* (Laveran, 1904). One adult *A. delalandii* specimen was collected from site 1 (25°27′43″S, 30°57′55″E), along the bank of the Crocodile River. Six adult *A. delalandii* were collected from site 2 (25°29′16″S, 30°59′32″E), a small stream flowing through a residential area. In 2018, a total of 25 *X. laevis* were collected from artificial ponds at the North-West University Botanical Gardens, Potchefstroom, South Africa (26°40′56″S, 27°05′43″E).

Blood was drawn from the femoral artery and smears were made onto microscope slides. Blood was also placed into a sterile 2 mL screw cap cryovial with 100% ethanol. The smears were fixed with 100% methanol and stained with a 10% Giemsa solution for approximately 20 min. Material from *A. delalandii* specimens in Potchefstroom was acquired from stored blood samples which had been collected during previous studies of Netherlands *et al*. (2014) and Conradie *et al*. (2017).

### Morphological characterization

Stained blood smears were screened and images were taken using Zeiss Axiocam 208 colour with a Nikon Eclipse Ni microscope and Zeiss Axiocam 305 colour with a Zeiss AX10 microscope at 1000× magnification. Images were measured using ImageJ version 1.52a (Schneider *et al*., 2012) according to a morphometric system adapted from Ferreira (2010) and Shannon (2016). Measurements include body length (BL); body width (BW) (measured at the point of maximum width, excluding the undulating membrane); nucleus length (NL); nucleus width (NW); undulating membrane width (UMW); number of undulations (NU); kinetoplast length (KL); kinetoplast width (KW); mid-nucleus to anterior body end distance (MA); mid nucleus to posterior body end distance (MP); free flagellum length (F). Body length (BL) of the round forms was measured between the 2 furthest apart points of the body. Body width (BW) of the round forms was measured at 90° to the BL. Derived measurements were: body shape index (BI) = BL/BW; nuclear index (NI) = NL/NW; nucleus position from the anterior end relative to body length, expressed as a percentage (NP) = MA/BL.

### DNA extraction, amplification and sequencing

DNA was then extracted from infected blood samples using a *Quick-*DNA™ Miniprep Plus Kit, according to the nucleated blood sample protocol (Zymo Research, California, USA). The extracted DNA samples were then transferred to a sterile 1.5 mL microcentrifuge tube and used in polymerase chain reaction (PCR) amplifications or stored when not in use at −20°C.

The 18S rRNA gene was targeted because it is currently the most commonly used gene for classifying anuran trypanosomes and hence has the most reference sequences available. Amplification of the gGAPDH (glyceraldehyde-3-phosphate dehydrogenase) gene was also attempted, however, was unsuccessful. Two overlapping fragments of the 18S rRNA gene were targeted for amplification using a nested PCR strategy adapted from McInnes *et al*. (2009) and Egan *et al*. (2020). The primary PCR was performed with the primers SLF (5′-GCTTGTTTCAAGGACTTAGC-3′) and S762.2 (5′-GACTTTTGCTTCCTCTAATG-3′), sourced from Inqaba Biotec. The secondary PCR was performed twice, using 2 different primer sets, namely B (5′-CGAACAACTGCCCTATCAGC-3′) and I (5′-GACTACAATGGTCTCTAATC-3′); and S825 (5′-ACCGTTTCGGCTTTTGTTGG-3′) and SLIR (5′-ACATTGTAGTGCGCGTGTC-3′), also sourced from Inqaba Biotec. The primary and secondary PCR cycle conditions included an initial denaturation step at 95°C for 5 min, annealing step at 50°C for 2 min and extension step at 72°C for 4 min. This was followed by 35 cycles comprising a denaturation step at 94°C for 30 s, annealing step at 55°C for 30 s and an extension step at 72°C for 2 min and 20 s. Lastly, a final denaturation step at 72°C for 7 min was performed. This entire amplification protocol was conducted on samples of *A. delalandii* (*n* = 7) from Mbombela and samples of *A. delalandii* (*n* = 10) and *X. laevis* (*n* = 10) from Potchefstroom. These specific samples were chosen, as they were found to be infected with trypanosomes during microscopic screening of the blood smears.

Electrophoresis with a 1% agarose gel was utilized to determine if the amplicons were of the correct size (±900 bp) and desired quality for sequencing. The samples that had clear bands and the correct size were sent to be sequenced with the chain-termination method at Inqaba Biotech (a commercial sequencing company). Samples of poor quality were discarded.

### Phylogenetic analysis

The chromatograph sequences (received from the sequencing at Inqaba Biotec) were edited and trimmed using FinchTV version 1.4.0. (Geospiza, 2006). A contig sequence was created for each sample from the forward and reverse primer sequences using the pairwise alignment method on BioEdit version 7.0.5.3 (Hall, 1999). The overlap of approximately 300 bp between B/I and S825/SLIR sequences was used to merge them and create final consensus sequences using BioEdit.

An nBLAST™ multiple alignment (McEntyre and Ostell, 2013) was performed on the final sequences to determine the per cent identity matches of the samples. Comparable 18S rDNA sequences, to be used for the construction of a phylogenetic tree, were obtained from NCBI GenBank with nBLAST™, whereas other sequences from the phylogenetic trees by Yazaki *et al*. (2017) and Bernal and Pinto (2016) were retrieved by accession number from NCBI GenBank. The sequence alignments were constructed using the MUSCLE alignment method on MegaX (Kumar *et al*., 2018) with default settings in FASTA format. The program jModelTest (Guindon and Gascuel, 2003; Darriba *et al*., 2012) was used to select the most suitable nucleotide substitution model for the sequence alignment with an Akaike information criterion calculation. The general time-reversible (GTR) model (Tavaré, 1986) with inverse (+I) and gamma (+G) distribution was selected as the best model, with a proportion of invariable sites 0.3190 and gamma shape of 0.4220. Maximum likelihood (ML) phylogenetic trees were constructed using RAxMLGUI 2.0 (Edler *et al*., 2021) with thorough bootstrap setting and 1000 bootstrap replicates. A Bayesian inference (BI) analysis was performed within MrBayes (Huelsenbeck and Ronquist, 2001; Ronquist and Huelsenbeck, 2003) using the GTR model with a proportion of invariable sites (+I) and a gamma-distributed rate variation (+G). This analysis used the Markov Chain Monte Carlo (MCMC) algorithm with 10 000 000 generations, where every 100th generation was sampled and the first 25% of the trees was discarded as burn-in. Due to the similar tree topology of ML and BI analyses, a consensus phylogram of the aquatic trypanosome clade was constructed and is represented on the BI tree. A model-corrected (GTR + I + G) pair-wise distances (*p*-distances) were calculated in PAUP version 4.0a169 (Swofford, 2003) for the sequences used in the phylogenetic analyses.

## Results

Of the 7 *A. delalandii* femoral blood samples collected from Mbombela, 1 (14%) was found to have trypanosomes conforming morphologically to *T. nelspruitense* during microscopic screening of the blood smears (sample AB200704A1). Additionally, trypanosomes with similar morphological characteristics to *T. nelspruitense* were also observed in 2 archived *A. delalandii* blood samples from Potchefstroom (samples AR150210A48 and AE150225B12). Three of 25 (12%) *X. laevis* from Potchefstroom exhibited trypanosomes conforming morphologically to *T. grandicolor* in the blood (samples AE180112A9, AE180112A6 and AE180112A5). Molecular sequencing was performed alongside morphological characterization for each sample due to the unreliability of only using morphological characteristics to classify trypanosomes.

### Diagnosis

Phylum: Euglenozoa Cavalier-Smith, 1981

Class: Kinetoplastea Honigberg, 1963, emend. Vickerman, 1976

Subclass: Metakinetoplastina Vickerman, 2004

Order: Trypanosomatida Kent, 1880

Family: Trypanosomatidae Doflein, 1951

Genus: *Trypanosoma* Gruby, 1843

Subgenus: *Trypanosoma* Gruby, 1843 emend. Votýpka and Kostygov, 2021

#### Redescription of *Trypanosoma* (*Trypanosoma*) *nelspruitense* Laveran, 1904 emend. Jordaan, du Preez and Netherlands

*Type host*: *Amietia delalandii* Duméril and Bibron, 1841 (Anura: Pyxicephalidae) (syns. *Amietia quecketti*, *Rana theileri* and *Rana angolensis*).

*Type material*: Hapantotype, 1× peripheral blood smear deposited in the Parasitic Worm Collection of the National Museum, Bloemfontein, South Africa under accession number NMB P 922 (AB200704A1). Other voucher material, 2× peripheral blood smears NMB P 924 (AR150210A48) and NMB P 923 (AE150225B12). Under Article 73.3.2 of the ICZN, the coinfection of *Hepatozoon theileri* (Laveran, 1905) in the hapantotype preparations is disclosed and excluded from the hapantotype.

*Type locality*: Mbombela (formerly Nelspruit), Mpumalanga province, South Africa (Laveran, 1904; Laveran and Mesnil, 1907).

*Localities in this study*: Stream in residential area (25°29′16″S, 30°59′32″E) Mbombela, Mpumalanga province, South Africa; Botanical Gardens (26°40′56″S, 27°05′43″E), North-West University campus, Potchefstroom, North West Province, South Africa.

*Site of infection*: Peripheral blood.

*Vector*: Unknown.

*Stages in vector*: Unknown.

*Representative DNA sequences*: The sequence data specifically associated with *T. nelspruitense* (upon which the present biological description is based) have been submitted to GenBank and are as follows: nuclear 18S rDNA (nu 18S) partial sequence OP502083, OP502084 and OP502085.

#### Description

**Fig. 1.**
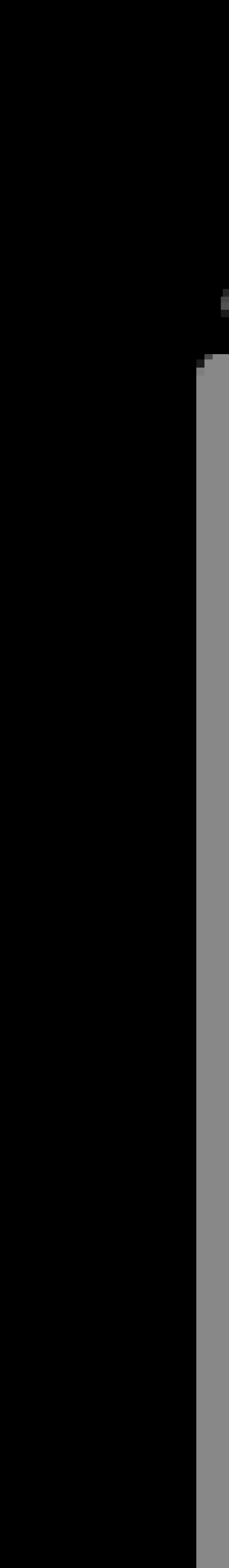
*Trypanosoma nelspruitense* plate. (A–H) Normal trypomastigote forms in the blood. (I, J) Round forms. (K) Stumpy form. (L) Larger normal form. Arrowheads show kinetoplasts (A–L); arrows show flagella (B and C) and undulating membranes (I and L); nuclei are indicated by ‘n’ (A, H, I, J and L). Internal flagella indicated by ‘f’ (I and J). Host cell indicated by ‘H’. Scale bar is 20 *μ*m.

Measurement range shown in *μ*m (mean ± standard deviation). Body length 46.68–55.82 (50.9 ± 3.28) and body width 4.19–10.69 (6.45 ± 1.67) (*n* = 18). Nucleus length 12.00–20.98 (14.9 ± 2.67) (*n* = 9); nucleus width 1.03–2.20 (1.44 ± 0.36) (*n* = 10); undulating membrane width 2.24–5.68 (3.43 ± 0.94); number of undulations 6–12 (8.78 ± 1.8); kinetoplast length 0.80–1.72 (1.06 ± 0.2) and kinetoplast width 0.38–0.74 (0.52 ± 0.1) (*n* = 18). Mid-nucleus-to-anterior-body-end distance 33.64–42.15 (37.73 ± 2.64) and mid-nucleus-to-posterior-body-end distance 10.27–16.09 (13.28 ± 1.8) (*n* = 11). Free flagellum length 17.33–32.83 (24.29 ± 6.61) (*n* = 6). Body index 4.37–12.15 (8.41 ± 2.24), nuclear index 6.38–12.95 (10.53 ± 2.15) and nuclear position as a percentage 66.75–79.75% (73.17 ± 3.57). The body stains purple in colour with uniform density (Fig. 1A–H). The undulating membrane stains lighter purple with a colourless and transparent outer edge. The nucleus stains light pink and is thin and elongated. It is positioned parallel to the body and present in the posterior half. The kinetoplast is distinct, stains deep pink in colour and is typically positioned close to the posterior end. The flagellum is faint and not always visible.

#### Remarks

The mean morphometric measurements of this species from Mbombela and Potchefstroom were all within 2 *μ*m of each other, which was less than the standard deviation. Therefore, and because the nucleotide sequences of both localities were identical, both localities’ specimen morphometrics were combined in Supplementary Table 1, as it is evident that they are the same species based on morphometric and molecular data. A small number of round and stumpy forms were observed alongside the normal morphotype for this species, as seen in Fig. 1I–K.

This trypanosome described in the present study infecting *A. delalandii* from Mbombela morphologically resembles the description of *T. nelspruitense* by Laveran (1904) from the same type host and type locality. It shared the characteristic long, slender body, long free flagellum and pronounced undulations. The body size of *T. nelspruitense* was larger yet remained proportionate to the dimensions in Laveran's (1904) description (50.9 × 6.45 *vs* 24–35 × 2.5–3.5 *μ*m). *Trypanosoma nelspruitense* has a distinctly free flagellum (24.29 *μ*m), which is in accordance with the size (20–35 *μ*m) described by Laveran (1904), where he also notes it as being ‘unusually long’. However, Laveran (1904) estimates its length to be generally the same length as the body, a 1:1 ratio, whereas the body length to free flagellum ratio in the present study was observed to be more than twice as long, at 2.1:1. There is no observed difference in stained colour or density between the posterior and anterior, in contrast to Laveran's (1904) description. This is possibly due to the visible colour or apparent density changing, depending on the staining method, although Giemsa solution (containing Azure, methylene blue and eosin) and methylene blue with eosin staining compounds were used in the present study and Laveran (1904), respectively.

The kinetoplast, which Laveran (1904) refers to as the centrosome, is stated as consistently being positioned slightly away from the posterior end, which is also true in the present redescription. Furthermore, the body posterior ends do not taper and are rounded, unlike the description of Laveran (1904). The variance in morphological measurements between the 2 parasites could also be due to the difference in techniques and microscopy equipment used between 1904 and 2022. Laveran (1904) states the parasite's dimensions are fairly consistent and the standard deviation is indeed shown to be low by this study. Only the s.d. of the free flagellum length of this species varied significantly which is likely due to the difficulty of accurately measuring it, as the full flagellum of the majority of specimens was obscured and often folded. *Trypanosoma nelspruitense* had a pronounced undulating membrane with a rounded up average NU of 9. The average NU is not stated by Laveran (1904), only that many of the specimens he encountered had 12 undulations, which is also true for *T. nelspruitense* in the present study. The nucleus of normal trypomastigote forms of *T. nelspruitense* is not rounded or oval as described by Laveran (1904), instead it is observed to be thin and elongated. Rounded nuclei were observed in the round and stumpy forms of *T. nelspruitense* (Fig. 1I–K). This difference could be due to the parasites being in different stages of division, as Ivanic (1936) reported an elongated nucleus in a ‘giant’ trypomastigote form and round nucleus in a round form of apparently the same species, *T. rotatorium*, stating that the protoplasmic body influences the nucleus shape. Diamond (1965) believes the situation described by Ivanic (1936) could be a mixed infection of 2 species of *Trypanosoma* and that the different nucleus forms could be due to different stages of nucleic division, although this is unproven.

Because of the advancements in technology and the addition of molecular profiles in species descriptions of protozoans since 1904, the original description of *T. nelspruitense* by Laveran (1904) may be considered incomplete by modern standards. Compounding matters further, there is no type material available for comparison. However, based on the data provided in the original description and for the reasons stated above, we propose the redescription and designation of a hapantotype of *Trypanosoma* (*Trypanosoma*) *nelspruitense* Laveran, 1904 emend. Jordaan, du Preez and Netherlands from the same type locality and type host as the original description. With this, we honour the legacy of Charles Louis Alphonse Laveran and his remarkable contribution to the knowledge of blood parasites.

In comparison with other trypanosomes from anuran hosts, normal trypomastigote forms of *T. nelspruitense* in the present study morphologically resemble those of the ‘larger’ form of *T. karyozeukton*, described by Dutton and Todd (1903) infecting west African anurans. *Trypanosoma karyozeukton* is, however, longer (67.2 *μ*m), has a shorter free flagellum (15.2 *μ*m) and has a characteristic chain of granules which is absent in *T. nelspruitense*; furthermore, there is a great geographic distance between these 2 species’ localities and the host species are different. *Trypanosoma nelspruitense*, from the present study, also shares morphological similarities with experimentally infected ‘adult’ forms of the anuran trypanosome *T. pipientis* (see Figs 5 and 6, Diamond, 1965). However, *T. pipientis* was shorter and narrower (43.4 × 3.4 *μ*m) than *T. nelspruitense*, and is geographically restricted to North America, infecting different host species. Furthermore, these experimentally infected forms of *T. pipientis* might not be morphologically representative of a natural infection.

Variation in the normal trypomastigote form was observed for *T. nelspruitense* (Fig. 1L). A small number of specimens were noticeably larger and stained faintly with a light pink colour, in contrast to the typical deep purple. Many of these larger specimens had damaged or poorly defined outer body membranes making their measurement imprecise. These larger specimens were not different enough to be distinguished as a separate morphotype from the normal form and were thus included in the morphometric measurements, when not damaged. Stumpy (Fig. 1K) and round forms (Fig. 1I and J) were sometimes observed alongside the *T. nelspruitense* infections which are believed to be a different morphotype of *T. nelspruitense* and not a different species. A single round form of *T. nelspruitense* in this study (Fig. 1J) had similar features to *T. chattoni* reported by Diamond (1965) and Shannon (2016), where the kinetoplast appears to be intranuclear, and an internal flagellum is observed. The other rounded form observed (Fig. 1I) also has an internal flagellum visible starting at the kinetoplast; however, the nucleus is positioned separately, and the formation of an undulating membrane is visible.

*Subgenus*: *Haematomonas* Mitrophanow, 1883 emend. Votýpka and Kostygov, 2021

#### Redescription of *Trypanosoma* (*Haematomonas*) *grandicolor* Pienaar, 1962 emend. Jordaan, du Preez and Netherlands

*Type host*: *Xenopus laevis* Daudin, 1802 (Anura: Pipidae).

*Type material*: Hapantotype, 1× blood smear from the type-host *X. laevis*, deposited in the Parasitic Worm Collection of the National Museum, Bloemfontein, South Africa under accession number NMB P 927 (AE180112A9). Other voucher material deposited, 2× blood smear from *X. laevis*, deposited in the Parasitic Worm Collection of the National Museum, Bloemfontein, South Africa under accession numbers NMB P 925 (AE180112A5) and NMB P 926 (AE180112A6).

*Type locality*: Stream (‘spruit’), Potchefstroom, North West province, South Africa.

*Localities in this study*: North-West University Botanical Gardens (26°40′56″S; 27°05′43″E), North-West University campus, Potchefstroom, North West province, South Africa.

*Site of infection*: Peripheral blood.

*Vector*: Unknown.

*Stages in vector*: Unknown.

*Representative DNA sequences*: The sequence data specifically associated with *T. grandicolor* (upon which the present biological description is based) have been submitted to GenBank and are as follows: nuclear 18S rDNA (nu 18S) partial sequence OP502081 and OP502082.

#### Description

**Fig. 2.**
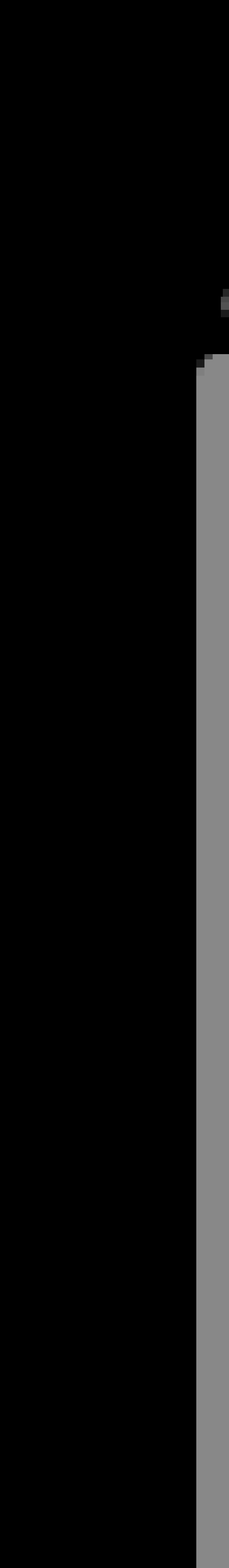
*Trypanosoma grandicolor* plate. (A–K) Normal trypomastigote forms in the blood. (L) Larger normal form. Arrowheads show kinetoplasts (A–L); arrows show flagellum (F) and undulating membranes (H and L); nuclei are indicated by ‘n’ (B, I and L). Scale bars are 20 *μ*m.

Measurement range shown in *μ*m (mean ± standard deviation). Body length 84.39–151.38 (111.73 ± 13.13) (*n* = 30); body width 9.57−26.19 (16.49 ± 4.41) (*n* = 33); nucleus length 4.99–15.13 (9.1 ± 2.4) (*n* = 32) and nucleus width 3.05–7.18 (5.34 ± 1) (*n* = 32); mid-nucleus-to-anterior-body-end distance 51.17–79.12 (61.68 ± 6.33) (*n* = 30); mid-nucleus-to-posterior-body-end distance 34.56–69.73 (49.81 ± 8.45) (*n* = 32); undulating membrane width 1.22–4.47 (2.18 ± 0.76) (*n* = 15); number of undulations 12–19 (15.69 ± 2.09) (*n* = 16); 1.01–1.74 (1.37 ± 0.18) and kinetoplast width 0.69–1.22 (0.94 ± 0.13) (*n* = 31). Free flagellum length 1.80–5.93 (3.68 ± 1.44) (*n* = 6). Body index 3.1–12.51 (7.26 ± 2.14) (*n* = 31); nuclear index 1.13–3.38 (1.76 ± 0.47) (*n* = 32) and nuclear position as a percentage 51.05–66.04% (55.56 ± 3.91) (*n* = 31). Body stains dark purple in colour with uniform density (Fig. 2A–K). The undulating membrane stains lighter purple when visible with a colourless and transparent outer edge, cutting across the parasites body (see Fig. 2A–L). The nucleus is ellipsoid in shape, staining light purple and is positioned centrally to the body. The kinetoplast is small, stains deep pink in colour and is typically positioned in halfway between the nucleus and posterior end. The flagellum is faint and not always visible.

#### Remarks

Morphologically, the trypanosome from the present study is large and basophilic in shape, with a prominent undulating membrane, and short flagellum visible. This species conforms morphologically to *T. grandicolor*, a species previously reported from South Africa by Pienaar (1962). However, the species reported by Pienaar (1962) was poorly described without any type material deposited.

Only 1 trypomastigote form was observed in this study as well as by Pienaar (1962). One especially large specimen was observed (Fig. 2L); however, it appears as if this form is partially damaged, as also seen with the large form of *T. nelspruitense* (Fig. 1L). The measurement ranges for the body length and width of the species of *Trypanosoma* given by Pienaar (1962) are 120–150 × 13–16 *μ*m, respectively. In the present study, morphometric ranges of the species of *Trypanosoma* are 84.39–151.38 × 9.57–26.19 *μ*m, conforming with the species of *Trypanosoma* from Pienaar (1962) and falling within the ranges of a giant anuran trypanosome, as it is larger than 50 *μ*m in length. The nucleus is indeed observed to be stained lighter with an average nuclear position of 55.56%, similar to the position of approximately 3/5^ths^ from the anterior body end as described by Pienaar (1962). In contrast to Pienaar (1962), the kinetoplast is clearly visible in the present study and is positioned approximately one-third of the body length from the posterior end. The kinetoplast is positioned towards the posterior at the base of the undulating membrane. The nucleus is oval with an average nuclear index (NL:NW) ratio of 1.7:1. Some granules are observed throughout the body, not just the anterior end as with Pienaar (1962) and the body stains evenly, with longitudinal striations observed that run its entire length. Based on the above descriptions, we redescribe *Trypanosoma* (*Haematomonas*) *grandicolor* Pienaar, 1962 emend. Jordaan, du Preez and Netherlands in the present study, found parasitizing the host *X. laevis*. Additionally, we designate a hapantotype from the same type host and type locality as the original description of *T. grandicolor*.

*Trypanosoma grandicolor* is classified as belonging to the giant anuran trypanosome complex (≥50 *μ*m in length) (Martin *et al*., 2002). This species is morphologically similar to the reptilian trypanosome, *T. superciliosae*, although the former can be differentiated by its larger body size, circular nucleus and shorter flagellum. Furthermore, the infection of *T. superciliosae* (Walliker, 1965) is reported in a different class of host from a separate continent. Trypomastigotes of *T. schmidti* (see Figs 55 and 56, Diamond, 1965) bear a morphological resemblance to *T. grandicolor*; however, the former species is both shorter and narrower, with a longer flagellum. Although similar, *T. superciliosae* has a shorter BL and BW (96.2 and 14.2 *μ*m) than *T. grandicolor* and has an elongated nucleus instead of the elliptical nucleus found in the latter species. Additionally, the flagellum of *T. superciliosae* is over 4 times longer on average (16.1 *μ*m) and the posterior was reported to stain paler than the rest of the body, which is in contrast to *T. grandicolor* from the present study. *Trypanosoma superciliosae* is also geographically isolated from *T. grandicolor*, as it was described from the iguanomorph lizard *Uranoscodon superciliosus* (syn. *U. superciliosae*) from Brazil. *Trypanosoma schmidti*, an anuran trypanosome from Florida, USA, also shares morphological similarities with *T. grandicolor*; however, it only has a BL of 85.9 and BW of 9.3 *μ*m, which are roughly 30 and 77% smaller than the latter species, respectively (Diamond, 1965). The flagellum of *T. schmidti* reportedly measures 13.6 *μ*m long which is 3.7 times longer than that of *T. grandicolor*. The host species and geographic locality of *T. schmidti* are also distinct from *T. grandicolor*. Notably, *T. schmidti* was described from experimentally infected frogs, and might not represent the wild phenotype occurring in natural infections.

### Molecular characterization

A multiple alignment BLAST™ was performed to determine the per cent identity matches of the samples’ sequences. All 3 samples of *T. nelspruitense*, OP502083, OP502084 and OP502085, were shown to be a single genotype, due to their close sequence matches. The sequences OP502083 and OP502084 had 100% identity matches for the query cover of 1356 bp (aligned using ClustalW), with a *p*-distance of 0.00 (Supplementary Table 2). Whereas the sequences OP502083 and OP502085 had 99.91% identity matches (differing by a single base pair) for their (ClustaIW) alignment with a query cover of 1223 bp, with a *p*-distance of 0.08. No matches above 96% were observed using BLAST™ for the 18S rDNA of *T. nelspruitense* and the closest sequence matches were from unnamed trypanosome species from South America with an interspecific divergence (model-corrected genetic distance) of 4.60.

**Fig. 3.**
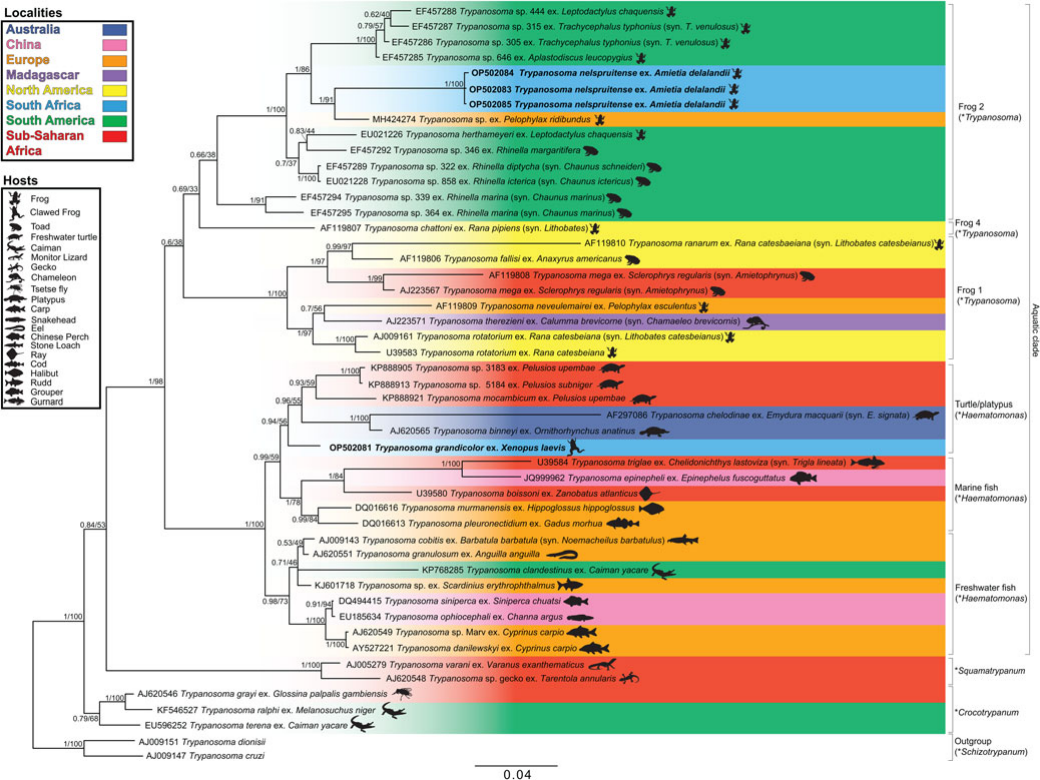
Consensus phylogram of the aquatic trypanosome clade based on 18S rDNA sequences. Tree topologies of maximum likelihood (ML) and Bayesian inference (BI) analyses were similar (represented on the BI tree). Nodal support values of BI posterior probability and ML bootstrap are shown as BI/ML. Sequences from the present study are in bold. Scale shown is nucleotide substitutions per site. Asterisk (*) indicates subgenus.

One *T. grandicolor* sequence, OP502082, was only a half fragment and was thus a shorter sequence (882 bp) than the other, OP502081 (1502 bp); however, the sequences shared 100% identity match, thus confirming they are the same species. The closest relatives to *T. grandicolor* are trypanosomes isolated from turtle, fish and platypus, with no matches above 98.1%, utilising BLAST™. Although the closest per cent identity match was shown by BLAST™ to be 98.06% with the freshwater fish trypanosome, *Trypanosoma cobitis*, with an interspecific divergence (model-corrected genetic distance) of 1.80, *T. grandicolor* was well-nested between species of freshwater turtle, platypus and marine fish hosts in the subgeneric clade *Haematomonas* of the phylogram in Fig. 3, with a BLAST™ identity match of 97.33% and interspecific divergence of 3.25 with *Trypanosoma* sp. 5184.

### Phylogenetic analysis

The consensus phylogram in Fig. 3 represents the aquatic trypanosome clade and is shown with few bootstraps below 75% and posterior probability below 0.80, indicating a high level of confidence. The clades with lower bootstrap values are likely caused by a deficiency of aquatic trypanosome sequences, making it difficult to position the clade precisely. The relationship of the clades in this study's phylogram is consistent with that of other studies’ phylogenies and is believed to be an accurate representation of part of the larger trypanosome phylogeny (Lima *et al*., 2012; Dvořáková *et al*., 2015; Attias *et al*., 2016; Bernal and Pinto, 2016; Spodareva *et al*., 2018; Kostygov *et al*., 2021). The *Crocotrypanum* and *Squamatrypanum* clades are shown to be separate from the aquatic clade as expected, the turtle/platypus clade is positioned with a close relation to the fish clades, and there is a split between the anuran (clade frog 2 and 4) and anuran/chamaeleon (clade frog 1) trypanosomes. This division appears to be geographically related, as there appears to be a dichotomy between North American and South American species. However, trypanosomes of other geographic localities are interspersed between the 2 anuran clades.

The present study focused on the aquatic clade. The trypanosome species from this study, *T. nelspruitense* (GenBank Accession OP502083, OP502804 and OP502085) and *T. grandicolor* (GenBank Accession OP502081 and OP502082), are placed in 2 separate novel monophyletic subclades, confirming their identity as 2 distinct species. The 2 South African trypanosomes from this study do not clade with any other African anuran species. There is a significant estimated evolutionary distance between the *T. nelspruitense* clade and its nearest hypothesized clade, the unnamed South American anuran trypanosomes. It possibly indicates that the common ancestral lineage of these 2 groups could have existed during the period of Gondwanaland and diverged evolutionarily with the break-up of this land mass into the continents of South America and Africa. *Trypanosoma grandicolor* is grouped within a clade of the platypus, turtle and marine fish trypanosomes. It clades closely with African turtle trypanosomes, *T. mocambicum* and sequences of unnamed trypanosome species from terrapins in Mozambique. This relatedness is likely due to the neighbouring distribution of South Africa and Mozambique, their aquatic hosts and potential leech vector transmission.

## Discussion

### Morphological characterization

Morphological analysis is notoriously unreliable with anuran species of *Trypanosoma* due to their lack of rigid structures and extreme pleomorphism (Shannon, 2016; Spodareva *et al*., 2018). Some species of *Trypanosoma* are known to have several different morphotypes and have a widespread distribution (Desser, 2001). However, morphology can still aid in distinguishing species by being used in conjunction with molecular analyses. Two distinct species were found in this study by analysing blood smear slides. Different forms for the same species were also observed to be present. There are no morphological measurements available for closely related African anuran trypanosome species from other studies to compare against the measurements of specimens in this study.

Suspected pleomorphism was observed on the same slides as the ‘normal’ trypomastigote morphotypes for both species. No dividing stages were observed in any of the blood samples. It is believed that these forms are not separate from their associated species, as some species of *Trypanosoma* are known to have multiple morphotypes associated with different stages of their life cycles. Interestingly, most of the polymorphism is seen within the leech and dipteran vectors of anuran trypanosomes (Netherlands, 2019; Vanhove *et al*., 2022); however, there are several reports of rounded trypanosome forms. Shannon (2016) found round forms of *T. chattoni* in the peripheral blood of North American species of *Lithobates*. Interestingly, *T. chattoni* is only known to occur as rounded forms in the vertebrate host and not the typical trypomastigote form (Diamond, 1965). Fantham *et al*. (1942) also found rounded forms of *T. rotatorium* mostly in the organs (spleen, heart, liver and lung) and less frequently in the peripheral blood of 2 species of anurans (*X. laevis* and *A. fuscigula*) from South Africa, as well as several Canadian and European anurans. Diamond (1965) is of the opinion that these rounded forms of Fantham *et al*. (1942) are not conspecific with *T. rotatorium*, and instead belong to *T. chattoni*, although it cannot be determined as no description or figure was provided in the original report. These forms were additionally reported to have different morphological changes such as an absent undulating membrane and flagellum. Laveran and Mesnil (1907) confirmed this morphological variance in several examples with the occurrence of ovoid and rounded forms of several species of *Trypanosoma* in frogs and visually established that it is a normal trypanosome that changed shape, which they believed to be due to the change in conditions when leaving blood vessels [and not a different species as suggested by Mayer (1843) and Grassi (1881) (see Laveran and Mesnil, 1907)]. These forms are described by Laveran and Mesnil (1907) and Fantham *et al*. (1942) as sometimes lacking an undulating membrane and/or flagellum, as can be seen with some forms of *T. nelspruitense* in Fig. 1I and J. Future studies focusing on the life cycle and identifying morphotypes in the vector could elucidate the different developmental stages for each species. A culturing method might be useful in studying the different forms; however, trypanosomes from cultures are often known to have extremely variable morphology and are not necessarily representative of the wild type (Ferreira *et al*., 2007).

### Molecular characterization

The method used in this study can present problems when sequences are isolated from host blood samples with infections of multiple species of *Trypanosoma*. Having a larger sample size could possibly resolve this dilemma by increasing the potential number of hosts with an infection of only 1 trypanosomatid species, resulting in a single morphospecies per sequence. Dvořáková *et al*. (2015) used PCR-based screening methods to determine the species of samples with mixed infections. The manner of DNA isolation used in the present study is a ‘catch-all’ method, isolating the trypanosomal DNA from the frog's but not from other trypanosomes in the sample. In the future, it might be possible to isolate individual trypanosome specimens from the blood sample and extract only their genetic material.

### Phylogenetic analysis

Trypanosomatid phylogenies can often be misleading due to the multi-host life cycle of trypanosomes, which cause overlap and merging of seemingly unrelated clades (Martin *et al*., 2002). This indistinct separation of trypanosome clades is compounded by the fact that most of these vector-transmission routes are unknown. The dipteran and leech vectors of trypanosomes are typically generalist feeders and can potentially transmit trypanosomes between unrelated animal taxa. Trypanosomes are generally transmitted through dipteran or leech vectors. These vector organisms are usually not host-specific and target many different types of species. Theoretically, the same species of *Trypanosoma* could be found in fish, amphibian and reptilian hosts, with vectors such as mosquitoes and leeches transmitting infections between the various host species. This poses the question: is there an overlap of the trypanosome clades’ hosts or a trypanosome species that links 2 clades (e.g. amphibian and squamate)? Spodareva *et al*. (2018) consider the amphibian trypanosomes to be a potential link between the aquatic and terrestrial clades, as they are transmitted by both leech and dipteran vectors.

In future, it could be beneficial to implement additional statistical methods of constructing phylogenetic trees and compare trees of different DNA barcodes, such as gGAPDH, to see if the hypothesized evolutionary relationships stay consistent across the different comparative methods. However, this would require other studies’ co-operation in obtaining enough trypanosome sequences using these DNA barcodes for comparison. Increased sampling of trypanosome sequences would allow us to get closer to a representation of the true trypanosomatid phylogeny and eventually bridge the gaps between the current phylogeny's clades.

## Conclusion

The morphological analysis proved useful in addition to the molecular analyses within the present study, as 2 distinct morphological species were supported in the phylogeny with 2 discrete taxonomic units. Larger sample sizes or improved DNA isolation methods could provide more certainty about the genetic identity of morphological species in future studies. Based on the combination of molecular and morphological data, redescriptions of 2 species of *Trypanosoma* infecting South African anurans are presented in this study: (1) *T. nelspruitense* Laveran, 1904, which was recorded from infected *A. delalandii* hosts from Mbombela and Potchefstroom; and (2) *T. grandicolor* Pienaar, 1962, recorded from infected *X. laevis* hosts from Potchefstroom. No discernible difference between the molecular and morphological data of *T. nelspruitense* from Potchefstroom and Mbombela was observed, showing it is the same species which occurs in multiple localities. Due to the morphological plasticity of trypanosomes and occurrence of mixed species infections in a single host, additional studies would be helpful in providing further insight and perspective. Ultimately, this study is just a starting point for the taxonomic resolution of the anuran trypanosome clade and creates a platform for future taxonomic characterization of trypanosomes. Further research needs to be conducted in order to grasp the true ecology and phylogeny of anuran trypanosomes.

## Data Availability

Sequence data are available from NCBI GenBank, under accession numbers OP502081–OP502085.

## Supplementary material

For supplementary material accompanying this paper visit https://doi.org/10.1017/S0031182023000203. click here to view supplementary material
